# Photomethanation of Gaseous CO_2_ over Ru/Silicon Nanowire Catalysts with Visible and Near‐Infrared Photons

**DOI:** 10.1002/advs.201400001

**Published:** 2014-11-25

**Authors:** Paul G. O'Brien, Amit Sandhel, Thomas E. Wood, Abdinoor A. Jelle, Laura B. Hoch, Doug D. Perovic, Charles A. Mims, Geoffrey A. Ozin

**Affiliations:** ^1^ Materials Chemistry Research Group, Department of ChemistryUniversity of Toronto80 St. George StreetTorontoOntarioM5S 3H6Canada; ^2^ Department of Chemical Engineering and Applied ChemistryUniversity of TorontoOntario200 College St.TorontoM5S 3E5Canada; ^3^ Department of Materials Science and EngineeringUniversity of Toronto184 College Street.TorontoOntarioM5S3E4Canada

**Keywords:** solar fuels, photocatalysis, silicon nanowires, photochemical catalysis, thermochemical catalysis

## Abstract

**Gaseous CO_2_ is transformed photochemically and thermochemically** in the presence of H_2_ to CH_4_ at millimole per hour per gram of catalyst conversion rates, using visible and near‐infrared photons. The catalyst used to drive this reaction comprises black silicon nanowire supported ruthenium. These results represent a step towards engineering broadband solar fuels tandem photothermal reactors that enable a three‐step process involving i) CO_2_ capture, ii) gaseous water splitting into H_2_, and iii) reduction of gaseous CO2 by H_2_.

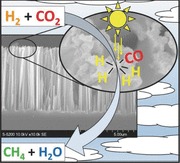

The vast majority of energy consumed by the human population is derived from burning fossil fuels because of their abundance and remarkably high energy density. However, the environmental and economic consequences of burning fossil fuels are well known. For every ton of burned carbon 3.67 tons of greenhouse gas CO_2_ emissions are released into the atmosphere and CO_2_ concentrations reached almost 40 Gt in 2013.^1^ From an economic standpoint, erratic prices related to increasing the supply of oil to match demands have a negative effect on the economy.^2^, ^3^

The radiant solar energy impinging on the earth's surface over one hour is greater than the world's annual energy usage and an alternative solution to the impending energy and climate crises is solar fuels.^4^, ^5^, ^6^, ^7^, ^8^, ^9^ The concept of solar fuels is based on harnessing an abundant supply of energy from the sun and storing it in the form of chemical bonds. The most common solar fuel investigated in the literature is hydrogen gas generated from solar powered water splitting.^10^, ^11^, ^12^, ^13^, ^14^ Other solar fuels reactions involving the reduction of CO_2_ to generate carbon‐based fuels such as carbon monoxide (CO), methane (CH_4_), and methanol (CH_3_OH) offer another source of energy with neutral CO_2_ emissions. Here, we investigate the photoreduction of CO_2_ over a Ru catalyst supported by silicon nanowires (Ru/SiNW) in a hydrogen environment. We consider this solar assisted CO_2_ conversion as a complementary solar fuels reaction that can potentially use a renewable source of hydrogen to simultaneously reduce greenhouse gas emissions and provide methane to natural gas pipeline networks 15, 16 (see Figure S1, Supporting Information).

Gas phase photomethanation of CO_2_ in a hydrogen environment was initially reported using a catalyst composed of dispersed Ru‐RuO_*x*_ on TiO_2_.^17^ Enhanced methanation rates were originally attributed to the chemical effects of electron–hole pairs generated from UV‐light absorption in the TiO_2_ support. However, subsequent studies revealed that photo­active species adsorbed on the catalyst surface^18^ as well as the increased temperature of the catalyst under light irradiation^19^ played a more significant role in increasing the methanation rates rather than the direct band‐gap absorption of the TiO_2_ support. Since this initial study, numerous catalysts have been tested for photoactivated CO_2_ reduction with H_2_. For example, Yoshida et al. tested TiO_2_, ZrO_2_, V_2_O_5_, Nb_2_O_5_, Ta_2_O_5_, WO_3_, ZnO, and MgO and found that of these materials, only ZrO_2_ and MgO exhibited photoactivity for the reduction of CO_2_ to CO in a H_2_ atmosphere.^20^, ^21^ Lo et al. also demonstrated the photoreduction of CO_2_ with H_2_ over ZrO_2_ in a circulating photocatalytic reactor.^22^ More recently, CO_2_ photoreduction to methanol in a hydrogen environment has been reported to occur over graphene oxide^23^ and zinc‐copper‐gallium layered double hydroxide catalysts.^24^ Also, very recently Hoch et al. have shown that hydroxylated indium oxide nanoparticles are active for the photoreduction of CO_2_ to CO.^25^

In general, when testing catalysts for the photoactive reduction of CO_2_ it is important to ensure that the products do not originate from adventitious carbon sources.^26^ In this context, isotope tracing experiments using Fourier transform infrared (FTIR) spectroscopy and mass spectroscopy are particularly effective.^5^ Further, it is interesting to note that CO_2_ photo­reduction rates reported in the literature for catalysts tested using isotope tracing experiments are on the order of 1 μmol gcat^‐1^ h^‐1^ or less. However, very recently this trend was broken when Sastre et al. reported the complete photocatalytic reduction of CO_2_ to methane in a hydrogen environment using a catalyst comprised of Ni on a silica‐alumina support.^27^ The complete methanation of CO_2_ reported in this work infers a CO_2_ photoreduction rate well over 10 mmol gcat^‐1^ h^‐1^. It was proposed that the reaction mechanism involves photogenerated electrons (holes), reducing (oxidizing) H_2_ to form Ni‐H, which then functions as the active CO_2_ reducing agent. Moreover, by performing experiments with optical filters it was determined that 76% of the photoreduction of CO_2_ was activated using UV light, which is consistent with the photon energy required to excite electrons across the 3.8 eV bandgap of NiO.^28^ It is also noteworthy that this proposed mechanism is reinforced by previous experiments reporting the methanation of CO_2_ over NiO‐based catalysts that were pretreated in an H_2_ atmosphere under UV‐light.^29^

Here, we report the photomethanation of gas‐phase CO_2_ over Ru nanoparticles sputtered onto black silicon nanowire (SiNW) supports in a hydrogen environment. SiNWs are particularly attractive as a support for solar powered catalysis because, with a band‐gap of 1.1 eV, they can potentially absorb 85% of the solar irradiance.^30^ Moreover, when vertically etched into a Si wafer these supports exhibit minimal reflection over a broad spectral range, and such wafers are often referred to as “black silicon”.^31^ Photomethanation rates over our Ru/SiNW catalyst are on the order of 1 mmol gcat^‐1^ h^‐1^ when normalized to the weight of the Ru, and we expect these rates can be greatly increased by optimizing the Ru nanoparticle dispersion over the SiNWs and using solar concentration. Moreover, regarding the chemical reaction mechanism, experimental results provided herein show that these Ru/SiNW catalysts photoactivate the Sabatier reaction both thermochemically and photochemically.^32^ That is, from a thermochemical standpoint, the Ru/SiNW catalyst heats up when irradiated with solar‐simulated light and methanation rates increase due to increased temperatures. Additionally, regarding photochemical activation, we show that at a set temperature, the rate of the Sabatier reaction increases proportionally to the number of incident photons with energy greater than the band‐gap of Si. Based on this strong experimental evidence we propose that a small fraction of photogenerated electrons and/or holes in the Si support facilitate the formation of active hydrogen atoms that participate in the overall photomethanation reaction. Further, it is noteworthy that while only a small fraction of photogenerated charge carriers contribute to the photomethanation reaction over the Ru/SiNW catalyst, we demonstrate that the photochemical contribution to the overall reaction rate is significant and greater than the thermochemical contribution under concentrated solar‐simulated irradiation.

Black SiNWs were prepared using the metal‐assisted chemical etching (MaCE) technique.^33^ Control samples were prepared on Corning Eagle XG glass and polished Si wafers, which were first cleaned in piranha solution. Subsequently, approximately 10 nm of Ru was sputtered onto the SiNW, glass and polished Si substrates to form the Ru/SiNW, Ru/Glass and Ru/Si catalysts, respectively. Scanning electron microscopy (SEM) images of the polished Si, Eagle XG glass, and SiNW with Ru sputtered onto their surfaces are shown in **Figure** **1**. A cross‐sectional SEM image of the Ru/SiNW catalyst is also shown in Figure 1, and the SiNWs are about 100 nm in diameter and approximately 6 μm in length. The sputtered Ru resides primarily at the top of the SiNWs and no Ru is present at the base of the SiNWs next to the Si wafer. The absorption spectra for these Ru/SiNW, Ru/glass and Ru/silicon catalysts are shown in Figure 1e. The absorption of the Ru/glass sample increases gradually from about 20% at *λ* = 300 nm to 40% at *λ* = 2500 nm. The absorption occurs almost entirely in the Ru as the absorption of the glass sample is low over this spectral region. The absorption of the Ru/Si sample is a bit higher than that of the Ru/glass sample, but follows a similar trend. There is also a dip in the absorption spectrum of the Ru/Si sample at the absorption edge of the Si wafer around 1100 nm. The Ru/SiNW catalysts absorbs 97% or more over the spectral region *λ <* 1000 nm and then drastically decreases to less than 70% at *λ* = 1200 nm. Beyond *λ* = 1200nm the absorption of the Ru/SiNW catalyst decreases steadily to a value of ≈40% at *λ* = 2500 nm. The Ru/SiNW catalyst is highly absorbing compared to the Ru/glass and Ru/Si samples because, as shown in Figure S2 (Supporting Information), it exhibits low reflection losses over the entire solar spectrum.

**Figure 1:**
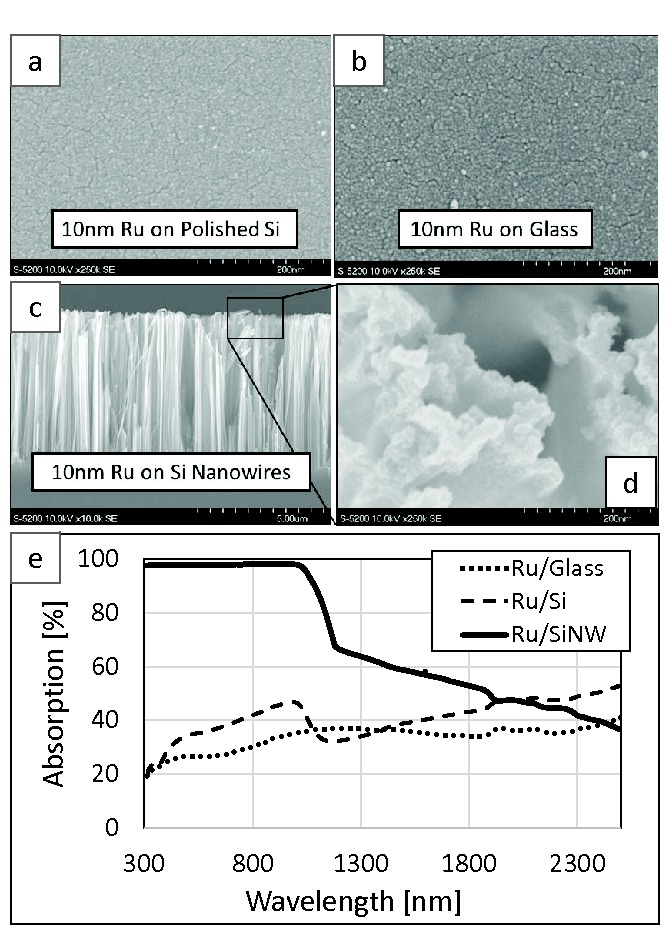
SEM image of 10nm of Ru sputtered onto a) a polished silicon wafer, b) a glass substrate, and c,d) silicon nanowires. Images (a,b,d) were acquired at 250 000 magnification while the cross‐sectional SEM image shown in (c) was acquired at a magnification of 10 000. e) The absorption spectra for the polished Si, glass and SiNW supports are plotted as a function of wavelength.

We initially tested the photoactivity of the Ru/SiNW, Ru/glass and Ru/Si catalysts at a temperature of 150 °C under solar simulated light from a Xe lamp over a duration of 6 h. The lamp intensity was 3.2 suns and the irradiated area of each sample was 1 cm^2^. The H_2_:CO_2_ gas ratio was 4:1 at a pressure of 45 psi and the results are plotted in **Figure** **2**a. The Sabatier reaction proceeded at a rate of 6.18 × 10^−2^ mmol g^‐1^ h^‐1^ over the Ru/glass sample in the dark and 7.52 × 10^−2^ mmol g^‐1^ h^‐1^ when irradiated with the Xe lamp. CO_2_ methanation rates over the Ru/Si sample increased by 84% from 7.44 × 10^−2^ mmol g^‐1^ h^‐1^ in the dark to 0.14 mmol g^‐1^ h^‐1^ in the light. However, CO_2_ methanation rates were the highest over the Ru/SiNW catalyst, proceeding at a rate of 0.51 mmol g^‐1^ h^‐1^ in the dark and increasing by 94% to 0.99 mmol g^‐1^ h^‐1^ in the light. Furthermore, these methanation rates were confirmed using ^13^CO_2_ isotope tracing experiments (see Figure S3, Supporting Information). Using isotope tracing experiments we also showed that bare SiNW without Ru were not active towards the Sabatier reaction (results not shown). Having verified the photoactivity of the Ru/SiNW catalyst at 150 °C, a second set of experiments were carried out to investigate the activity of this catalyst when exposed to solar simulated light without supplemental heating from an external source. That is, we disconnected the heater and monitored the temperature of the Ru/SiNW catalyst under the Xe lamp at an intensity of 14.5 suns. Moreover, to gain insight regarding the degree of conductive and convective cooling from the gaseous reactants we performed batch reaction tests using a H_2_:CO_2_ gas ratio of 4:1 at 15, 30 and 45 psi and the temperature profiles over the three hour duration of these tests are plotted in Figure 2b. For each run the temperature of the sample increases rapidly at the beginning of the test when the Xe lamp was turned on and then continues to rise gradually over the duration of the reaction until the lamp is switched off at the 3 h point. It is also noteworthy that the sample temperature of the reactions run at 15, 30, and 45 psi reach a maximum temperature of 125 °C, 117 °C, and 107 °C, respectively. As to be expected, raising the reactor pressure increases the amount of conductive and convective cooling thereby decreasing the maximum temperature attained by the Ru/SiNW catalyst. The CO_2_ methanation rates corresponding to the reactions carried out at 15, 30, and 45 psi are plotted in Figure 2c. While the CO_2_ methanation rates are comparable for the three different pressures, within about 20% of each other, a maximum methanation rate of 0.80 mmol g^‐1^ h^‐1^ is measured at a pressure of 30 psi. It is known that the Sabatier reaction rate increases with increasing pressure of the reactant gases, however as shown in Figure 2b, in going from a pressure of 30 psi to 45 psi the catalyst temperature drops by about 10 °C causing a decrease in reaction rates. That is, higher concentrations of reactants at higher reactor pressures accelerates the reaction but also increases the amount of conductive and convective cooling which decreases the reaction rate. As a consequence of these two opposing effects, the highest reaction rates are observed at an intermediate pressure of 30 psi.

**Figure 2:**
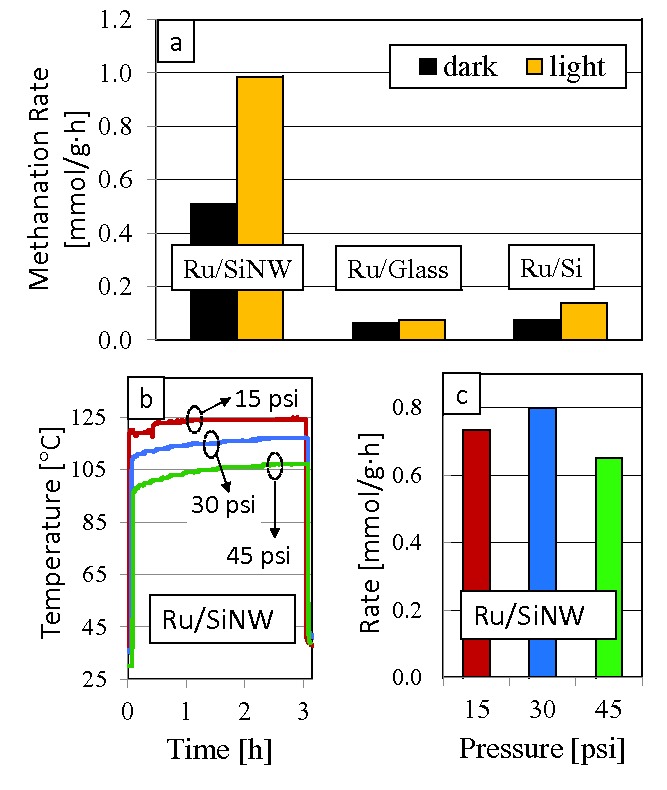
Methanation rates over Ru‐based catalysts on the SiNW, glass and polished Si supports at a) 150 °C and 45 psi. b) Temperature profiles recorded for batch reactions performed at 15, 30 and 45 psi and c) corresponding methanation rates. All tests were performed at a H_2_:CO_2_ gas ratio of 4:1. Note that the methanation rates are normalized to the weight of the Ru catalyst.

We also performed a set of experiments to measure the activation energy of the Ru/SiNW catalyst. The CO_2_ methanation rates over the Ru/SiNW catalyst in the dark are plotted as a function of temperature in **Figure** **3** and the inset shows that the corresponding activation energy is 54.5 kJ mol^‐1^. This is in agreement with the activation energy reported in the literature for the Sabatier reaction when carried out over Ru‐based catalysts.^17^ Furthermore, we also measured the effective activation energy under solar‐simulated radiation. Specifically, we disconnected the heater and performed a set of batch reactions with varying light intensities in order to measure the Sabatier reaction rates plotted as the yellow line shown in Figure 3. Using these photomethanation rates we then calculate an effective activation energy of 53.1 kJ mol^‐1^ as shown in the inset in Figure 3. Thus, while the Sabatier reaction rates are greater under solar‐simulated radiation, the activation energy does not differ significantly whether heating *via* solar‐simulated radiation or a resistive heating source.

**Figure 3:**
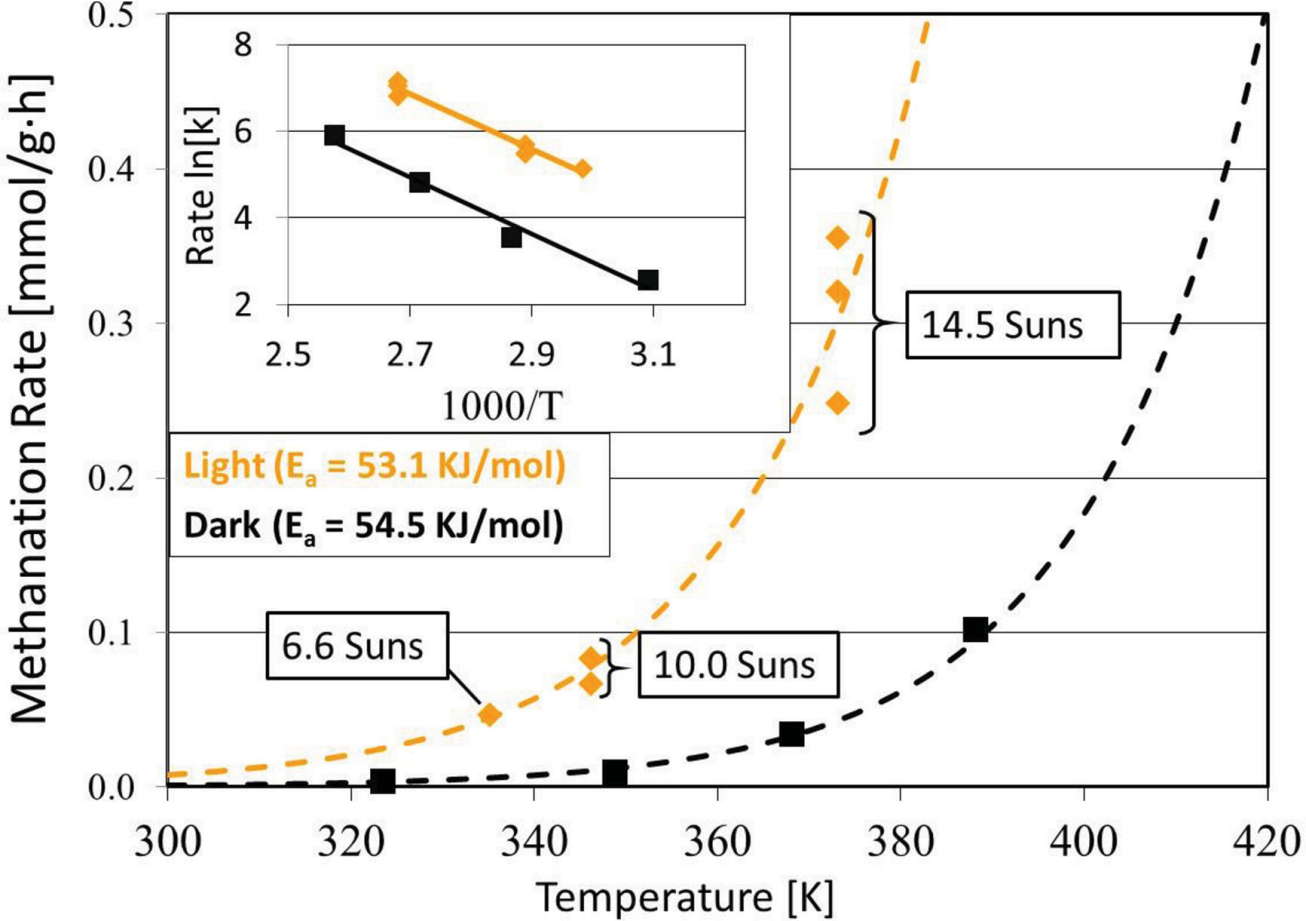
Methanation rates plotted as a function of temperature in the dark (black) and under solar‐simulated irradiation (yellow). The line of best fit to an exponential function is shown for the series of batch reactions carried out in the light (dashed yellow line) and dark (dashed black line). The inset shows these methanation rates on a plot of ln(*k*) vs 1000/*T* used to calculate the activation energy over the Ru/SiNW catalyst in the light and dark. Note that the methanation rates are normalized to the weight of the Ru catalyst.

The fact that the activation energy is comparable, whether the Ru/SiNW catalyst is heated with solar simulated radiation or with a resistive heater, suggests that the reaction mechanism under irradiation is similar to that in the dark. However, since the reaction rates are higher under the Xe lamp, incident photons must cause some effect that accelerates the Sabatier reaction mechanism. To gain more insight into how the impinging photons accelerate the reaction rates we performed another set of experiments wherein the temperature was held constant while the incident photon flux impinging onto the Ru/SiNW catalyst was varied. This set of experiments included seven batch reactions all carried out at a temperature of 93 °C, the results of which are illustrated in **Figure** **4**. Specifically, as shown in Figure 4a, five of these seven tests, labelled A through E, were performed using high‐pass cut‐off filters such that for A: *λ* > 300 nm, B: *λ* > 495 nm, C: *λ* > 615 nm, D: *λ* > 715 nm, and E: *λ* > 850 nm. For each batch reaction the intensity of the Xe lamp was adjusted such that the temperature of the Ru/SiNW catalyst was always set to 93 °C. The photon distribution irradiating the Ru/SiNW catalyst for batch reactions A through E are shown in Figure 4b. Moreover, the total number of photons with energy greater than 1.1 eV (the bandgap of the Si support) for each of these batch runs is also provided in the inset in Figure 4a. Here it can be noted that the total number of photons impinging onto the sample increases as the cut‐off wavelength of the high‐pass filter decreases. This is because the average thermalization energy provided to the Ru/SiNW sample is smaller for longer wavelength photons and thus more photons are required to heat the sample to 93 °C. However, for cases D: *λ* > 715 nm, and E: *λ* > 850 nm when the Xe lamp was set to full intensity the sample reached a maximum temperature of 65 °C and 54 °C, respectively. Thus, for tests D and E, supplementary heating was supplied using the temperature controller such that the temperature of the Ru/SiNW catalyst was maintained at 93 °C for all reactions. The two other tests performed in the set of seven experiments include one test carried out in the dark and test F, wherein the Ru/SiNW catalyst was subjected only to sub‐bandgap photons with *λ* > 1100 nm. For test F the Ru/SiNW catalyst reached a temperature of 39 °C when subjected to the long wavelength radiation and supplementary heating was also provided in this case to increase its temperature to 93 °C.

**Figure 4:**
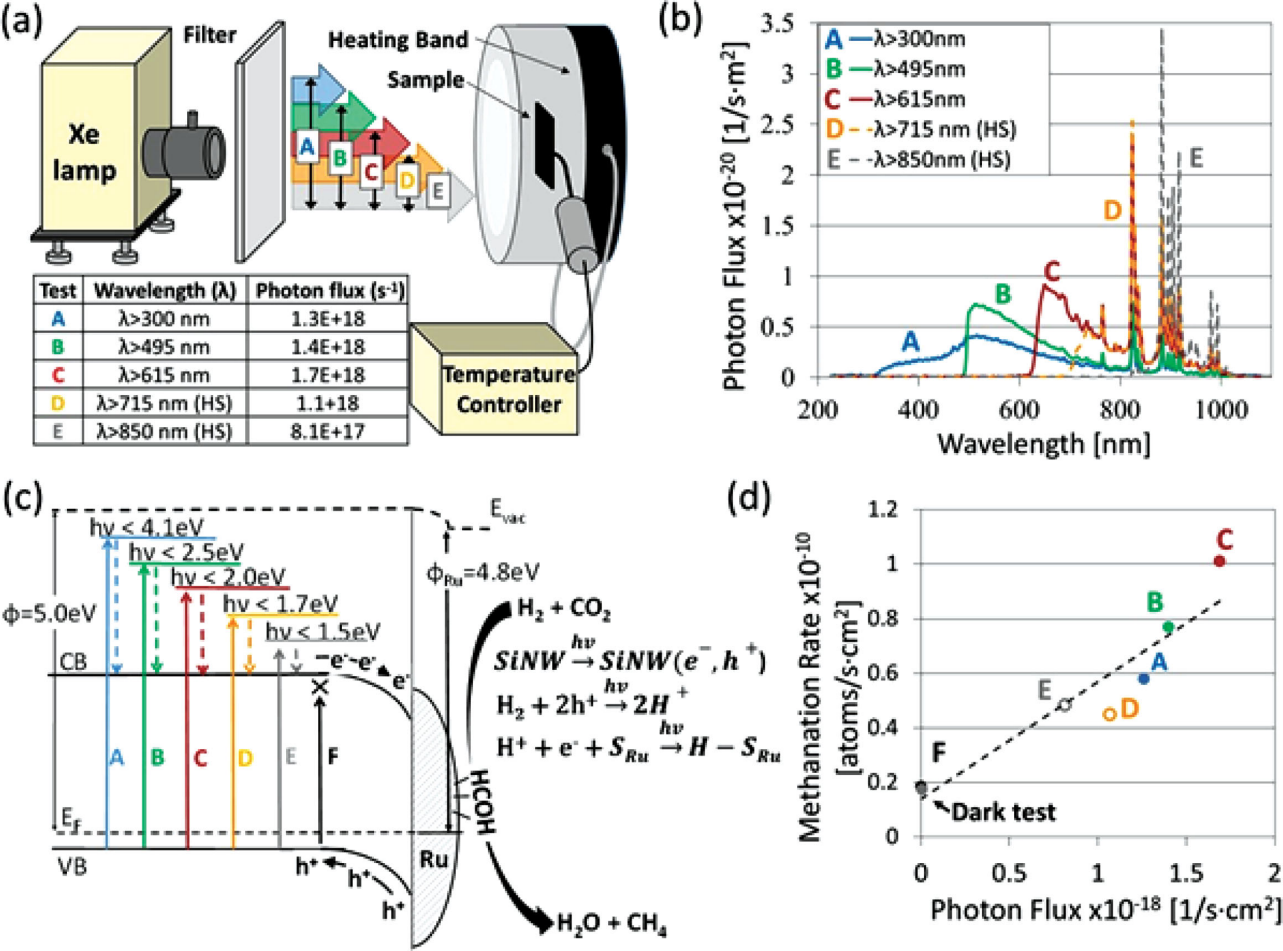
a) Schematic representation of experimental apparatus setup. A K‐type thermocouple was placed at the rear side of the Ru/SiNW catalyst, shielded from the incident light. For batch reactions D, E and F supplementary heating was provided to heat the catalyst to 93 °C. b) Spectra of photons with energy greater than 1.1 eV impinging onto the Ru/SiNW catalyst for batch reactions A through E. c) Energy band diagram at the SiNW‐Ru interface. A schematic diagram of the proposed reaction mechanism is also shown on the right. d) The Sabatier reaction rate is plotted as a function of the number of absorbed photons with energy greater than the bandgap of silicon for the seven batch reactions performed in this set of experiments in (d). The Sabatier reaction rates for the dark test as well as the test carried out under sub‐bandgap irradiation (test F) are both ≈0.2 molecules s^‐1^ cm^‐2^.

The Sabatier reaction rate is plotted as a function of the number of incident photons with energy greater than the bandgap of Si for the seven batch reactions performed in this set of experiments in Figure 4d. The methanation rate for the test carried out in the dark and for test F: *λ* > 1100 nm is about 2 × 10^9^ molecules per second. This suggests that sub‐band gap photons with energy less than the band‐gap of silicon do not activate the Sabatier reaction photochemically. Alternatively, we propose that heat generated from sub‐band gap photons absorbed in the Ru/SiNW sample activates the Sabatier reaction thermochemically, similarly to as if the heat was provided entirely from a thermal heating source. Figure 4d also shows that the Sabatier reaction proceeds five times faster when the Ru/SiNW sample is irradiated with photons in the spectral region 615 nm < *λ* < 1100 nm as compared to tests performed in the dark. Furthermore, when the Ru/SiNW catalyst is irradiated with photons in the near infrared spectral range (850 nm < *λ* < 1100 nm) the reaction rate is more than twice that of the dark reaction rate. Moreover, the slope of the line in Figure 4d is 4 × 10^−9^ CH_4_ molecules/photon and this suggests that only a very small fraction of incident photons induce photochemical activity in the Ru/SiNW catalyst. Thus, we can make the following two statements based on the results shown in Figure 4. 1) Photons with energy less than the band‐gap of silicon absorbed in the Ru/SiNW catalyst provide thermal energy that activates the Sabatier reaction thermochemically. 2) Photons with energy greater than the band‐gap of Si generate electron‐hole pairs in the Ru/SiNW. These excited charge carriers ultimately thermalize and recombine thereby producing heat that thermochemically accelerates the Sabatier reaction. Furthermore, a small fraction of incident photons with sufficient energy to excite electron‐hole pairs (EHPs) in the SiNW, on the order of ten out of every billion, photochemically activate the Sabatier reaction. Here it is important to note that while the photomethanation rates are proportional to only a small fraction of incident photons, the photochemical contribution to the overall Sabatier reaction rate is significant under concentrated solar‐simulated irradiation. In fact, in comparing batch reactions C and F in Figure 4d, or in comparing the yellow and black lines in Figure 3, it is apparent that the photochemical contribution to the reaction rate over the Ru/SiNW catalyst can be as much as 5 times greater than the thermochemical contribution. Furthermore, it should be noted that the photochemical contribution to the overall photomethanation rate on the Ru/SiNW catalyst is not observed for the Ru/glass catalyst (see Figure S4, Supporting Information). Moreover, similar experiments recently reported in the literature show that photomethanation reactions on Ru‐based catalysts with Al_2_O_3_ supports are driven photothermally and do not exhibit photochemical activity.^34^ The photochemical activity over the Ru/SiNW catalysts reported herein may be attributed to the lower band‐gap energy of silicon compared to other supports such as Al_2_O_3_ and may also be attributed to the quality of the interface between the Ru and the SiNW support formed during the vacuum‐based sputtering deposition.

There are a number of ways a small fraction of absorbed photons with energy greater than the bandgap of Si could photochemically enhance the Sabatier reaction rate over the Ru/SiNW catalyst. In this context, photochemical reaction mechanisms could involve localized heating or plasmons at the surface of the Ru particles,^35^ or photogenerated EHPs could influence the electronic polarity of the Ru/SiNW catalyst in a favorable manner.^36^ However, the Sabatier reaction has been studied extensively in the literature^37^ and is known to proceed thermochemically over Ru‐based catalysts at relatively low temperatures. In this context, and based on the comparable activation energies reported in Figure 3, we do not expect that the Sabatier reaction mechanism over the Ru/SiNW catalysts under illumination from the Xe lamp differs substantially from the thermochemical reaction mechanism that occurs in the dark. Rather, as discussed subsequently, we propose that photogenerated EHPs in the SiNW support accelerate the Sabatier reaction by activating adsorbed hydrogen atoms.

It is generally accepted that in the first step of the Sabatier reaction on Ru‐based catalysts CO_2_ readily dissociates to form adsorbed CO and O on the surface of Ru. The rate limiting step then involves the reaction of CO with 6 H atoms adsorbed on the Ru metal surface to form CH_4_ and H_2_O.^38^, ^39^, ^40^ While the exact reaction mechanism between the adsorbed CO and H atoms is still under debate it is clear that a large number of active H atoms in close proximity to reaction sites will enhance the Sabatier reaction rate. We expect that photogenerated EHPs assist with the formation of Ru‐H bonds that react with adsorbed CO to form CH_4_. An energy band diagram of a Schottky‐junction formed between the SiNW and Ru metal at the tip of the nanowire is shown in Figure 4c. The work function of Ru and electron affinity of Si were acquired from the literature.^41^, ^42^ The valence and conduction bands in the p‐type SiNWs bend down towards the Ru metal. Thus, electrons excited into the conduction band of the SiNW will be accelerated towards the Ru metal while holes generated in the SiNW see a potential barrier at the SiNW‐Ru interface. As shown in Figure 4c, we expect that H atoms interact with photogenerated holes (h^+^) to form H^+^ ions. These H^+^ ions can then interact with photogenerated electrons at the Ru surface to form Ru‐H bonds that participate in the overall Sabatier reaction. This process may involve tunneling of electrons from H atoms at the Ru surface into holes photogenerated in the SiNW or migration of protons to the Ru metal surface or both.

In conclusion, we have shown that Ru/SiNW catalysts prepared by sputtering small amounts of Ru onto SiNWs activate the Sabatier reaction both thermochemically and photochemically under solar‐simulated light. For example, the Ru/SiNW catalysts reported herein activates the Sabatier reaction at a rate of 0.74 mmol g^‐1^ h^‐1^ (Figure 2c) under 14.5 suns intensity of solar‐simulated irradiation in a hydrogen atmosphere at 15 psi and a H_2_:CO_2_ ratio of 4:1 when normalized to the weight of the sputtered Ru metal on the SiNW. One can expect to achieve much higher reaction rates by optimizing the dispersion of the Ru over the SiNW support. For instance, the sputtering process deposits the Ru only at the region near the upper surface of the silicon nanowires and the Ru dispersion can be improved by using other deposition processes such as wet impregnation, electrodeposition or atomic layer deposition. Moreover, it is expected that improving the Ru dispersion will significantly enhance photochemically induced reaction rates because electron‐hole pairs photogenerated along the length of the nanowires will have less distance to traverse and are less likely to recombine before reaching the active catalytic site at the Ru metal.

We have also shown that the photochemical effect can be induced using near‐infrared photons. Specifically, at 93 °C the Sabatier reaction rate is roughly doubled when the Ru/SiNW catalyst is irradiated with photons in the spectral range (850 nm < *λ* < 1100 nm). The ability of the Ru/SiNW catalyst to activate the Sabatier reaction using infrared photons has promising implications for the design of tandem solar fuels reactors that utilize the ultraviolet and visible portion of the solar irradiance to split water and generate H_2_, which can subsequently be used to reduce CO_2_ (see Figure S1, Supporting Information). In this context the potential to accelerate the Sabatier reaction is significant and optimal operating temperatures for driving the Sabatier reaction can be achieved using inexpensive parabolic trough solar concentrators.^43^ However, to put things into perspective, it should be noted that the rates reported herein (1 mmol/g·h) are still too low to reduce CO_2_ at globally significant rates (see Figure S1, Supporting Information). Further research is required to increase CO_2_ conversion rates over the Ru/SiNW catalyst reported herein and also to replace Ru with a less expensive catalyst such as Ni. Nevertheless, the discovery that CO_2_ can be reduced photochemically using a broad spectral range covering most of the solar spectrum is an important point to consider in designing solar fuels reactors. For example, rather than heating the entire reactor, solar radiation can be focused onto the catalyst in order to reduce the heating load and also to use available land‐space more efficiently. Furthermore, considering the Sabatier reaction rates as shown in Figure 3 as an example, a given reaction rate can be attained at a lower temperature by photochemically driving the reaction. This ability to achieve higher reaction rates at lower temperatures may produce numerous advantages. For example, lower operating temperatures may reduce the deleterious effects of sintering, poisoning, mechanical degradation and eventual deactivation of the catalyst.

As another point SiNWs can potentially be used as supports that provide heat from the sun to numerous other solar fuels catalysts loaded onto their surface to boost their reaction rates. For example, as a proof of concept experiment, we have shown that the reverse water gas shift can be activated over In_2_O_3_ nanoparticle photocatalysts loaded onto SiNW supports at ≈150 °C under irradiation from a Xe lamp (see Figure S5, Supporting Information). As a final point, SiNWs can be scaled to technologically significant proportions using well‐known silicon wafer wet‐chemistry processing. Thus, on account of their large surface area, high absorption towards solar irradiation, and technologically mature background, black SiNW supports merit further investigation in the pursuit and developement of practically useful gas‐phase solar fuels catalysts.

## Experimental Section

*Catalyst Fabrication*: Silicon nanowires were fabricated using a metal‐assisted chemical etching (MaCE) technique. p‐type silicon wafers were cut into 1 inch squares and then cleaned with ethanol, acetone and de‐ionized water. The wafers were further cleaned with piranha solution (H_2_SO_4_:H_2_O_2_ = 3:1 by volume) for 3 h and then rinsed with de‐ionized water. Subsequently, the wafers were immersed in an etching solution consisting of 5 M HF, 0.02 M AgNO_3_, and 3 mL of 10% HF solution in order to remove surface oxides. The solution is then placed in an autoclave and allowed to etch for 1 h at room temperature. After the etching process, silver dendrites covering the silicon nanowires were washed off with deionized water. To ensure all the silver nanoparticles and dendrites were removed the etched wafers were placed in concentrated nitric acid (18 M HNO_3_) for 30 min. The etched wafers were then washed and dried before being cut into 1cm^2^ pieces. Eagle XG and P‐type polished silicon wafers were cleaned in a solution of sulfuric acid/hydrogen peroxide (3:1 v/v) and then rinsed with distilled water. Ru was sputtered onto these samples which were subsequently cut into 1 cm^2^ squares. The deposition was carried out in a custom‐built sputtering system (Kurt J. Lesker Co.) by radio frequency (RF) magnetron sputtering using a 99.95% pure Ru sputtering target purchased from Angstrom Sciences, Inc. The base pressure of the sputtering chamber was pumped down to 1 × 10^−7^ Torr before argon was introduced into the chamber at a flow rate of 20 sccm. The chamber pressure was set to 3 mTorr during the deposition, which was carried out at room temperature. The forward power was 100 W and the substrate‐to‐target distance was 14 cm. The sputtering process was terminated when 10 nm of Ru, as measured from an in‐situ thickness monitor (SQM‐242 from Sigma), had been deposited.

*Characterization*: Absorption and reflection measurements were performed using a Lambda 1050 UV/VIS/NIR spectrometer from Perkin Elmer equipped with an integrating sphere with a diameter of 150 mm and a center mount holder. SEM images were taken using a Hitachi S‐5200 scanning electron microscope.

*Sabatier Reaction Rate Measurements*: Gas‐phase photocatalytic rate measurements were conducted in a custom‐built 1.5 mL stainless steel batch reactor with a fused silica view port sealed with Viton O‐rings. All samples were cleaned using the ZONE‐SEM cleaning system from Hitachi for 10 minutes prior to being loaded into the reactor. For heated tests the reactor temperatures were controlled by an OMEGA CN616 6‐Zone temperature controller combined with a thermocouple placed in contact with the sample. The pressure inside the reactor was monitored using an Omega PX309 pressure transducer. Product gases were analyzed with a flame ionization detector (FID) and thermal conductivity detector (TCD) installed in a SRI‐8610 Gas Chromatograph (GC) with a 3' Mole Sieve 13a and 6' Haysep D column. The thermocouple was placed at the front face of the sample and shielded from incident light unless otherwise specified. Isotope tracing experiments were performed using ^13^CO_2_ (99.9 atomic% Sigma Aldrich). The reactor was heated to 150 °C and purged with H_2_ for 10 min prior to being infiltrated with CO_2_ and H_2_ at a H_2_:CO_2_ ratio of 4:1. Isotope product gases were separated using a 60 m GS‐Carbonplot column and measured using an Agilent 7890A gas chromatographic mass spectrometer (GC‐MS). To measure the activation energy shown in Figure 3 the reactor was purged with H_2_ for 10 min prior to being infiltrated to a pressure of 45 psi with H_2_ and CO_2_ gas at a H_2_:CO_2_ ratio of 4:1. For tests carried out in the dark the reaction chamber was heated before purging with H_2_ gas. For tests wherein the sample was irradiated with light the lamp was turned on once the reactor valves were closed and the temperature of the sample was recorded over the duration of the test. A typical temperature profile shows the sample heating quickly at the beginning of the test when the light is turned on (see Figure S6, Supporting Information). The light intensity was varied between runs and the temperature recorded at the half‐way point of the test (at 90 min) was used in plotting Figure 3. Extra precautions were taken to ensure accuracy in measuring CO_2_ methanation rates as a function of the number of incident photons as plotted in Figure 4. The thermocouple was pressed firmly against the center of the back‐side of the SiNW sample. The sample was pre‐irradiated for 1 h in a pure H_2_ atmosphere at 45 psi prior to each test. The reactors were then infiltrated with CO_2_ and H_2_ gas. Manual valves were closed to seal the reactor from the infiltrating gases when the CO_2_ and H_2_ pressures had reached 3 psi and 12 psi, respectively. To be precise, the final partial pressure of CO_2_ and H_2_ in the reactor ranged from 3.0 to 3.2 psi and from 12.0 to 12.2 psi, respectively. This small variance in partial pressures may have produced some deviation from the linear trend for batch reactions A through F as plotted in Figure 4d (for example the reaction rate for batch reaction D is 6.9% less than that of E despite there being a greater incident photon flux for D). Nevertheless, despite this small variation in reactant partial pressures, the data presented in Figure 4d shows a strong linear correlation with a measure of goodness‐of‐fit of linear regression of *r* ^2^ = 0.90.The incident light spectra was varied between runs using high‐pass cut of filters. For each test using a different filter the intensity of the lamp was adjusted until the stabilized temperature of the sample was 93 °C. However, when the *λ* = 715 nm, *λ* = 850 nm, and *λ* = 1110 nm cut‐off filters were used the stabilized temperature of the sample was 65 °C, 54 °C, and 39 °C, respectively; for these cases supplementary heat was supplied using the OMEGA CN16 6‐Zone temperature controller until the sample reached 93 °C. The spectral output was measured using a StellarNet Inc spectrophotometer and the power of the incident irradiation was measured using a Newport 91150V calibrated reference cell and meter.

## Supporting information

As a service to our authors and readers, this journal provides supporting information supplied by the authors. Such materials are peer reviewed and may be re‐organized for online delivery, but are not copy‐edited or typeset. Technical support issues arising from supporting information (other than missing files) should be addressed to the authors.

Supplementary

## References

[1] James , A. Foley , Nat. World News, Nov. 19, 2013.

[2] J. Murray , D. King , Nature 2012, 481, 433 22281577 10.1038/481433a

[3] R. A. Kerr , Science 2011, 331, 1510. 21436416 10.1126/science.331.6024.1510

[4] C. A. Grimes , O. K. Verghese , S. Ranjan , Light, Water, Hydrogen: The Solar Generation of Hydrogen by Water Photoelectrolysis, Springer, Boston, MA 2003.

[5] Y. Izumi , Coord. Chem. Rev. 2013, 257, 171.

[6] S. Neatu , J. A. M. Agulló , H. Garcia , Int. J. Mol. Sci. 2014, 5246. 24670477 10.3390/ijms15045246 PMC4013561

[7] S. N. Habisreutinger , L. S. Mende , J. K. Stolarczyk , Angew. Chem. Int. Ed. 2013, 52, 7372. 10.1002/anie.201207199 23765842

[8] S. Navalón , A. Dhakshinamoorthy , M. Álvaro , H. Garcia , ChemSusChem 2013, 6, 562 23468280 10.1002/cssc.201200670

[9] S. Dasgupta , B. S. Brunschwig , J. R. Winkler , H. B. Gray , Chem. Soc. Rev. 2013, 42, 2213. 23426641 10.1039/c3cs90016a

[10] A. J. Cowan , J. R. Durrant , Chem. Soc. Rev. 2013, 42, 2281. 23023269 10.1039/c2cs35305a

[11] C. G. Morales‐Guio , S. G. Tilley , H. Vrubel , M. Grätzel , X. Hu , Nat. Commun. 2014, 5, 3059. 24402352 10.1038/ncomms4059

[12] S. C. Warren , K. VoÏtchovsky , H. Dotan , C. M. Leroy , M. Cornuz , F. Stellacci , C. Hébert , A. Rothschild , M. Grätzel , Nat. Mater. 2013, 12, 842. 23832125 10.1038/nmat3684

[13] J. Moir , N. Soheilnia , P. O'Brien , A. Jelle , C. M. Grozea , D. Faulkner , M. G. Helander , G. A. Ozin , ACS Nano 2013, 7, 4261. 23581965 10.1021/nn400744d

[14] K. S. Joya , Y. F. Joya , K. Ocakogly , R. van de Krol , Angew. Chem. Int. Ed. 2013, 52, 10426. 10.1002/anie.201300136 23955876

[15] G. A. Olah , G. K. S. Prakash , A. Goeppert , J. Am. Chem. Soc. 2011, 133, 12881. 21612273 10.1021/ja202642y

[16] J. A. de Gouw , D. D. Parrish , G. J. Frost , M. Trainer , Earth's Future 2014, 2, 75.

[17] K. R. Thampi , H. Kiwi , M. Grätzel , Nature 1987, 327, 506.

[18] C. Revilliod , A. J. McEvoy , M. Grätzel , Sol. Energy Mater. 1991, 24, 522.

[19] J. Melsheimer , W. Guo , D. Ziegler , M. Wesemann , R. Schlögl , Catal. Lett. 1991, 11, 157.

[20] S. Yoshida , Y. Kohno , Catal. Surv. Jpn. 2000, 4, 107.

[21] K. Teramura , T. Tanaka , H. Ishikawa , Y. Kohno , T. Funabiki , J. Phys. Chem. B 2004, 108, 346.

[22] C. Lo , C. Hung , C. Yuan , J. Wu , Sol. Energy. Mater. Sol. Cells 2007, 91, 1765.

[23] H. C. Hsu , I. Shown , H. Y. Wei , Y. C. Chang , H. Y. Du , Y. G. Lin , C. A. Tseng , C. H. Wang , L. C. Chen , Y. C. Lin , K. H. Chen , Nanoscale 2013, 5, 262. 23160369 10.1039/c2nr31718d

[24] M. Morikawa , N. Ahmed , Y. Yoshida , Y. Izumi , Appl. Catal. B 2014, 144, 561.

[25] L. B. Hoch , T. E. Wood , P. G. O'Brien , K. Liao , L. M. Reyes , C. A. Mims , G. A. Ozin , unpublished.

[26] C. C. Yang , Y. H. Yu , B. van der Linden , J. C. S. Wu , G. Mul , J. Am. Chem. Soc. 2010, 132, 8398. 20509650 10.1021/ja101318k

[27] F. Sastre , A. V. Puga , L. Liu , A. Corma , H. García , J. Am. Chem. Soc. 2014, 136, 6798. 24725054 10.1021/ja500924t

[28] R. J. Powell , W. E. Spicer , Phys. Rev. B 1970, 2, 2182.

[29] K. Ogura , M. Kawano , D. Adachi , J. Mol. Catal. 1992, 72, 173.

[30] K. Q. Peng , S. T. Lee , Adv. Mater. 2011, 23, 198. 20931630 10.1002/adma.201002410

[31] H. C. Yuan , V. E. Yost , M. R. Page , P. Stradins , D. L. Meier , H. M. Branz , Appl. Phys. Lett. 2009, 95, 123501.

[32] V. Balzami , F. Scandola , Energy Resources Through Photochemistry and Catalysis, (Ed: M. Grätzel), Academic Press Inc, New York 1983.

[33] X. Li , P. W. Bohn , Appl. Phys. Lett. 2000, 77, 2572.

[34] Meng , T. Wang , L. Liu , S. Ouyang , P. Li , H. Hu , T. Kako , H. Iwai , A. Tanaka , J. Ye , Angew. Chem. Int. Ed. 2014, 126, 11662. 10.1002/anie.201404953 25044684

[35] P. Christopher , H. Xin , S. Linic , Nat. Chem. 2011, 3, 467. 21602862 10.1038/nchem.1032

[36] S. Scirè , C. Crisafulli , R. Maggiore , S. Minicò , S. Galvagno , Catal. Lett. 1998, 51, 41.

[37] W. Wang , J. Gong , Fron. Chem. Sci. Eng. 2011, 5, 2.

[38] M. R. Prairie , A. Renken , J. G. Highfield , K. R. Thampi , M. Grätzel , J. Catal. 1991, 129, 130.

[39] S. Eckle , H. G. Anfang , R. J. Behm , J.Phys. Chem. C 2011, 115, 1361.

[40] F. Solymosi , A. Erdöhelyi , M. Kocsis , J. Chem. Soc. Farad. Trans. 1. 1981, 77, 1003.

[41] A. Novikov , Solid State Electron. 2010, 54, 8.

[42] H. L. Skriver , N. M. Rosengaard , Phys. Rev. B 1992, 46, 7157. 10.1103/physrevb.46.7157 10002423

[43] A. Fernández‐García , E. Zarza , L. Valenzuela , M. Pérez , Renew. Sust. Energ. Rev. 2010, 14, 1695.

