# Supplementary material for: Photomethanation of Gaseous CO2 over Ru/Silicon Nanowire Catalysts with Visible and Near‐Infrared Photons
Source: Adv Sci (Weinh). 2014 Nov 25;1(1):1400001. doi: 10.1002/advs.201400001 (PMC5115264; doi:10.1002/advs.201400001)
Supplement: Supplementary file 1 — Supplementary [file ADVS-1-0g-s001.pdf]

Copyright WILEY-VCH Verlag GmbH & Co. KGaA, 69469 Weinheim, Germany, 2014.

ADVANCED SCIENCE
Open Access

Supporting Information

for *Adv. Sci.*, DOI: 10.1002/advs. 201400001

Photomethanation of Gaseous CO_2 over Ru/Silicon Nanowire Catalysts with Visible and Near-Infrared Photons

Paul G. O'Brien, Amit Sandhel, Thomas E. Wood, Abdinoor A. Jelle, Laura B. Hoch, Doug D. Perovic, Charles A. Mims, and Geoffrey A. Ozin**

Supporting Information

Photomethanation of Gaseous CO_2 over Ru/Silicon Nanowire Catalysts with Visible and Near-Infrared Photons

Paul G. O'Brien, Amit Sandhel, Thomas E. Wood, Abdinoor Jelle, Laura B. Hoch, Charles A. Mims and Geoffrey A. Ozin

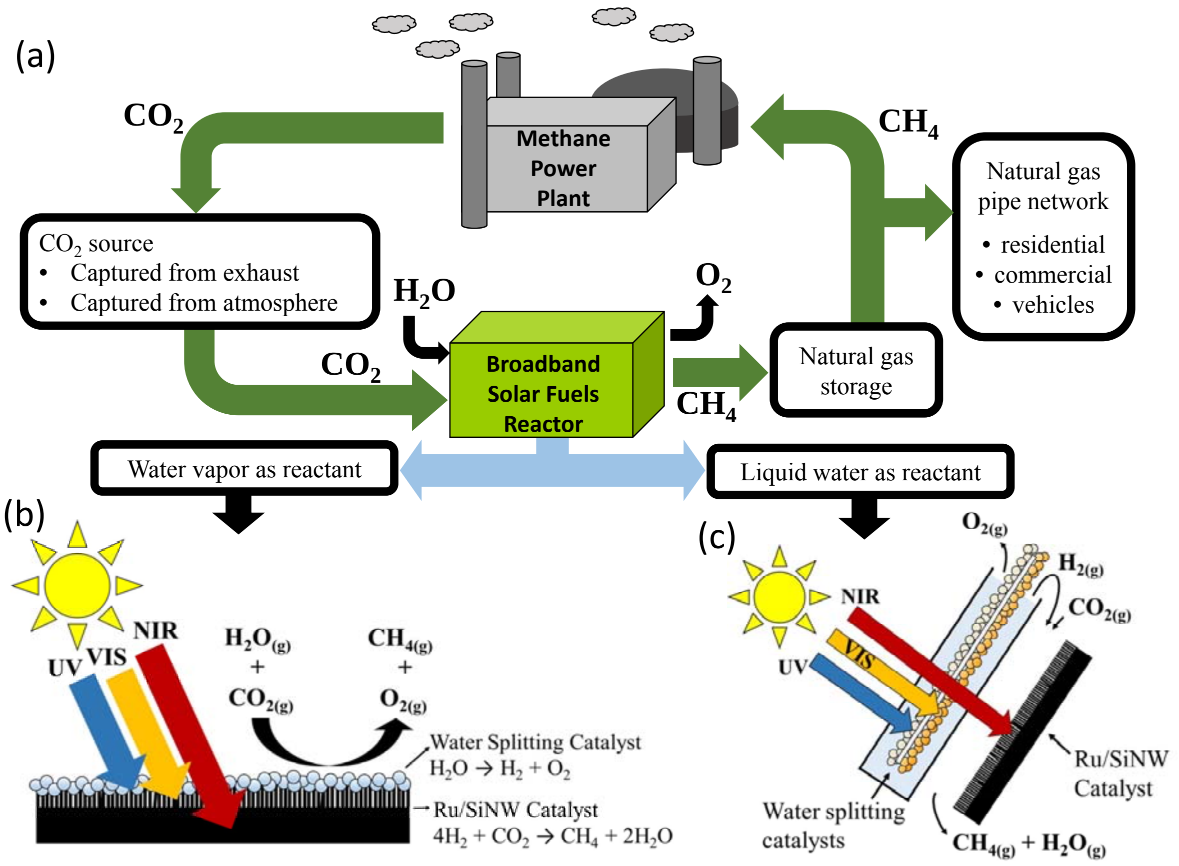

Figure S1 (a) Schematic diagram of a methane power plant and broadband solar fuels reactor integrated into a natural gas network cycle that recycles CO_2 to CH_4. The methane produced can be used for heating residential and commercial buildings and potentially transportation vehicles as well.[1] (b) A schematic diagram illustrating the idea of a thin catalyst film that utilizes UV- and visible photons to split water can be deposited on top of the Ru/SiNW catalyst. Hydrogen generated from the water-splitting reaction can be provided to the Ru/SiNW catalysts. NIR photons transmitted through the water-splitting catalyst can then be used to activate the Sabatier reaction over the Ru/SiNW catalyst. (c) The Ru/SiNW catalyst

could also be placed below a reactor that uses UV- and Visible photons to generate hydrogen from liquid water. In this tandem configuration the generated hydrogen can be exposed along with CO_2 across the surface of the Ru/SiNW catalyst. NIR photons transmitted through the reactor can then activate the Sabatier reaction over the Ru/SiNW catalyst. Also, here it should be noted that while the above schematic portrays an interesting concept for a solar fuels reactor, the initial rates reported for the Ru/SiNW catalyst (1 mmol/g·h) are still too low to reduce CO_2 at globally significant levels. To put things into perspective, it should be noted that even if these rates were increased by 3 orders of magnitude by optimizing the dispersion of the Ru over the SiNW it would still take ~10 tonnes of Ru to convert 1 Gt of CO_2 annually (annual human CO_2 emissions are ~40 tonnes).

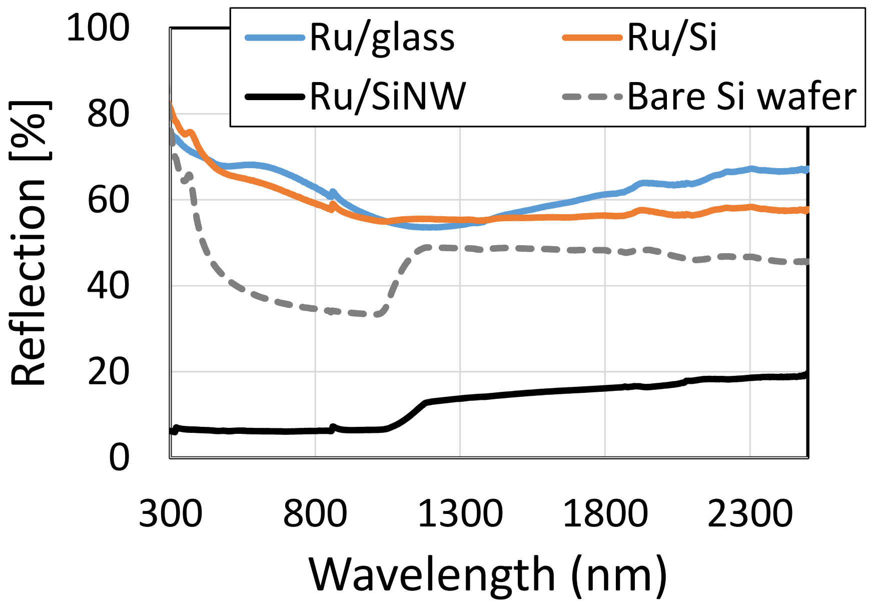

Figure S2 Reflectance spectra for catalysts comprised of ~10nm of Ru sputtered onto glass (blue line) polished silicon (orange) and silicon nanowire (black line) supports. The reflectance spectra from a bare polished Si wafer is also shown for comparison.

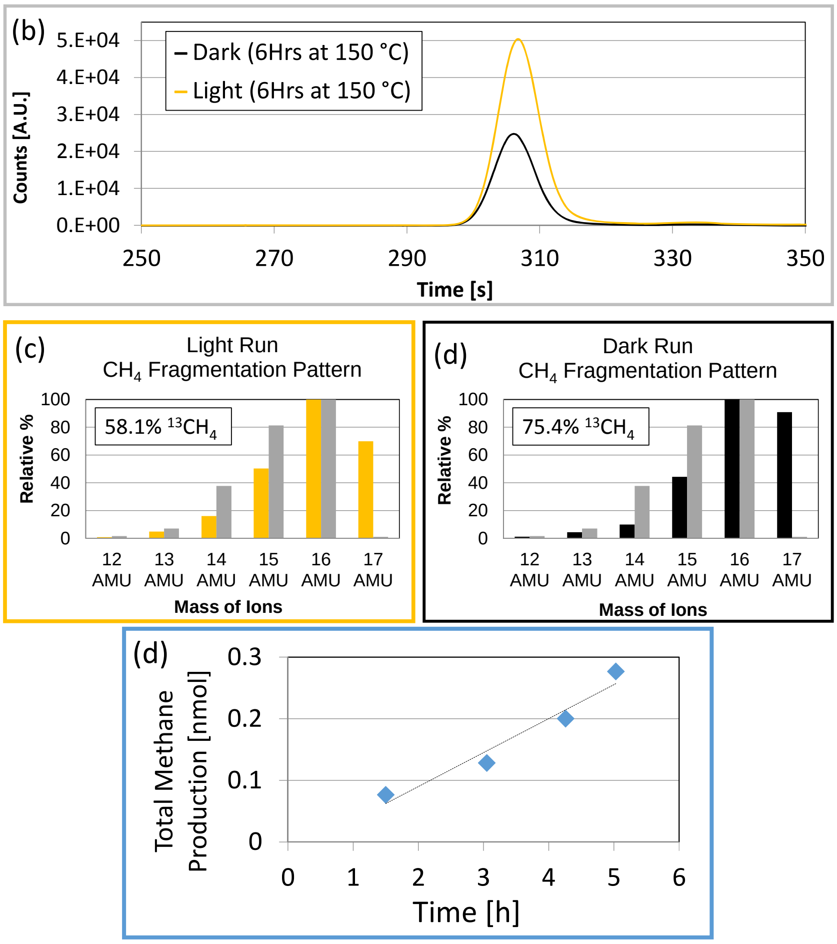

Figure S3 (a) Sabatier reaction rates for a sequence of eleven tests carried out on a Ru/SiNW catalyst at a temperature of 150°C. Multiple tests were performed to verify reproducibility. The tests are labelled chronologically as T1 through T11 and are listed from left to right. Tests carried out under solar simulated irradiation from the Xe lamp are shaded yellow while tests carried out in the dark are black. Methanation rates are initially high for a fresh sample (i.e. 2.69 mmol/g·h for T1) but level off to a steady value roughly between 0.78 and 0.98 mmol/g·h after the first few tests. The last two tests (T10 and T11) were carried out using isotope labelled $^{13}CO_2$ and 0.51 mmol/g·h of CH_4 was generated in the dark (T10) while 0.99 mmol/g·h was generated under the Xe lamp (T11). The fragmentation patterns acquired using an Agilent 7890A gas chromatographic mass spectrometer (GC-MS) for T10 and T11 are shown as Figures (b) and (c), respectively. These fragmentation patterns show that of the methane generated for the dark run (T10), 75% is $^{13}CH_4$, while 58% of the methane generated in the light run (T11) is $^{13}CH_4$. Thus, the total amount of $^{13}CH_4$ generated for the dark and light runs are 0.38 mmol/g·h for T10 and 0.57 mmol/g·h for T11, respectively. Furthermore, it is noteworthy that a large portion of the $^{12}CH_4$ generated in T10 and T11 likely originated from ^{12}CO that was adsorbed on the surface of the Ru/SiNW catalyst at the beginning of the

test. For example, isotope tracing experiments have shown that it takes as long as 1000 minutes to replace ^{12}CO adsorbed on Ru-based catalysts with its isotope labeled ^{13}CO counter-part.[2] Thus, it is likely that a good portion of the carbon in the $^{12}CH_4$ generated in tests T10 and T11 originated from CO_2 introduced in earlier tests. (Note that the methanations rates are normalized to the weight of the Ru catalyst). (d) We also performed a series of tests to show the time-evolution of the photomethanation rates over the Ru/SiNW catalyst even at low temperatures. Specifically rates are plotted in Figure (d) for a series of 4 batch reactions performed over a Ru/SiNW catlyst under ~10suns solar-simulated radiation without any external heating. The duration of the batch reactions was 1.5h, 3h, 4h and 5h. There is a strong linear correlation between the total amount of methane generated and the duration of the reaction (goodness-of-fit of linear regression of r^2=0.95).

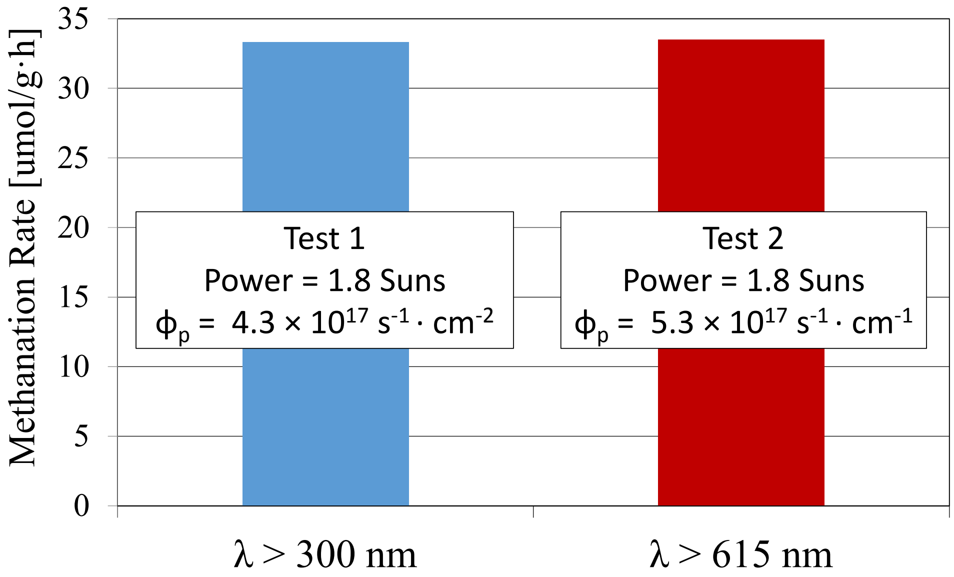

Figure S4 Photomethanation rates for the Ru/Glass catalyst under filtered irradiance from a 300W Xe lamp at a temperature of ~90°C. For test one (left) the wavelength of the incident photons is 300nm or greater while for test two (right) the wavelength of the incident photons is longer than 615nm. The irradiant power for both tests is 180 mW·cm^{-2}, while for tests one (left) and two (right) the photon flux, φ_p, is 4.3 x 10^{17} s^{-1}·cm^{-2} and 5.3 x 10^{17} s^{-1}·cm^{-2} respectively. The rates for the two tests are nearly identical, showing that the methanation reaction rates depend on the power of the incident radiation rather than the number of impinging photons. This suggests that photomethanation proceeds thermochemically rather than photochemically on the Ru/Glass sample. This is in contrast to the Ru/SiNW catalyst, which exhibits photomethanation rates that are proportional to the impinging photon flux as

shown in Figure 3. (Note that the methanations rates are normalized to the weight of the Ru catalyst).

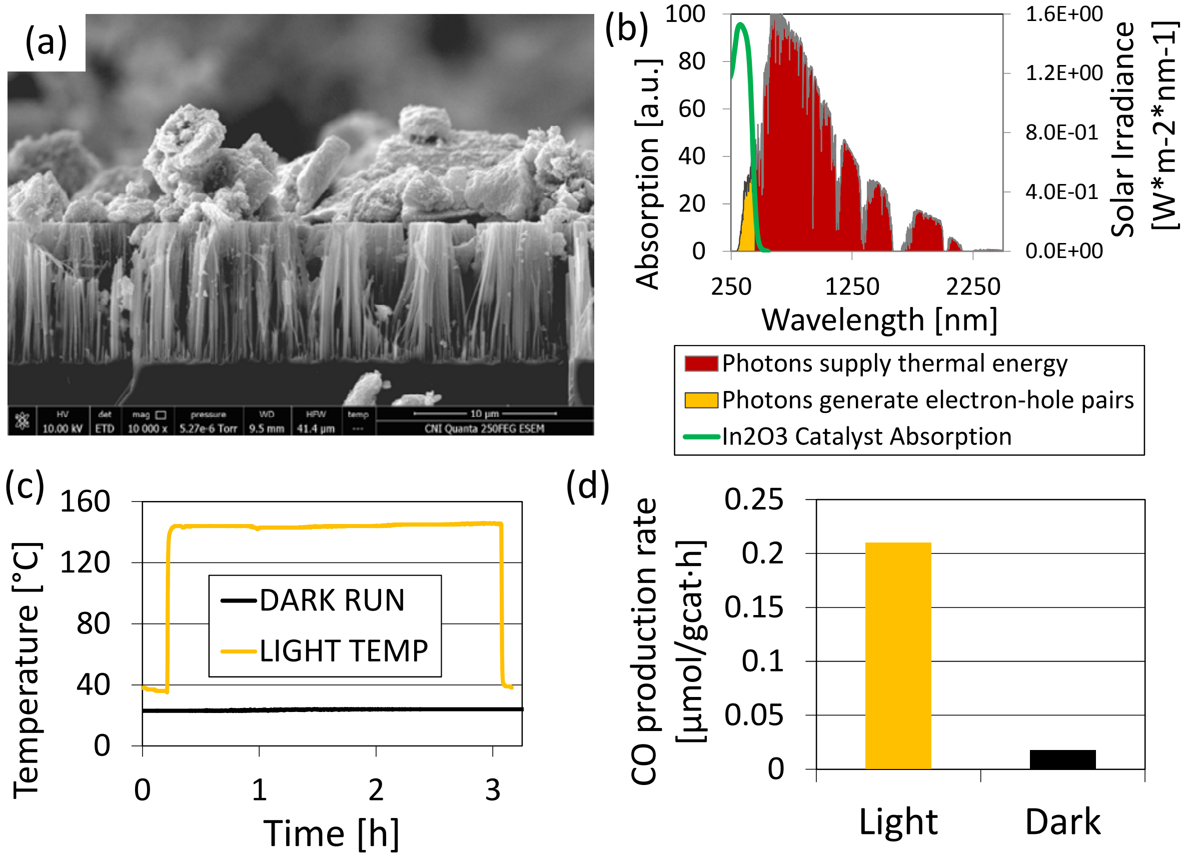

Figure S5 (a) Cross-sectional SEM image of In_2O_3 nanoparticle catalysts drop-cast onto SiNW supports (In_2O_3/SiNW catalyst). It has recently been reported in the literature that these In_2O_3 nanoparticle catalysts are active towards the reduction of CO_2 to CO at an optimal temperature of approximately 150°C.[3] The relative absorption spectra of In_2O_3 nanoparticle catalysts (green line) superimposed over top of the AM1.5 solar irradiance[4] is shown in (b). The yellow colored area of the solar spectrum represents the portion of the solar irradiance that can photochemically activate the In_2O_3 nanoparticle catalysts while the red colored area represents solar energy that can be used to provide thermal energy to heat the catalyst. In this proof of concept experiment we ran batch reaction tests to show that the In_2O_3 nanoparticle photocatalysts could be activated entirely by radiant energy without an external heating source. That is, we increased the light intensity irradiated from the Xe source to > 15 Suns and sub-band gap photons (red colored area in (b)) provided enough energy to heat the SiNW support to ~ 145°C while incident photons in the UV- and Vis- portion of the solar spectrum (yellow shaded area) photochemically activated the reaction. The temperature profile over the

duration of the 3 hour tests (measured with a thermocouple pressed against the rear side of the SiNW sample) carried out in the dark and under the Xe lamp is shown in (c). The rate at which CO is generated for these light and dark reactions is shown in (d). CO_2 reduction rates for the light run are greater than 0.2μmol/gcat·h. This rate is in good agreement with the rates reported in the literature, although the intensity of the Xe lamp was about 2 Suns. We expect that a more intense light source was required to drive the In_2O_3/SiNW catalyst because the temperature of the In_2O_3 nanoparticles was likely significantly less than the SiNW support. That is, as shown in (a), the In_2O_3 nanoparticles reside as clumps on the upper surface of the SiNWs and we expect that thermal heat transfer resistance through the In_2O_3 nanoparticles prevents them from reaching the same temperature of the SiNWs. Heat transfer to the In_2O_3 nanoparticles can be improved by increasing the contact area between the nanoparticles and the SiNW support. Nevertheless, this simple proof of concept experiment demonstrates that catalyst supports can be designed to use the broadband solar spectrum to simultaneously provide thermal energy and high energy photons that photochemically activate CO_2 reduction reactions.

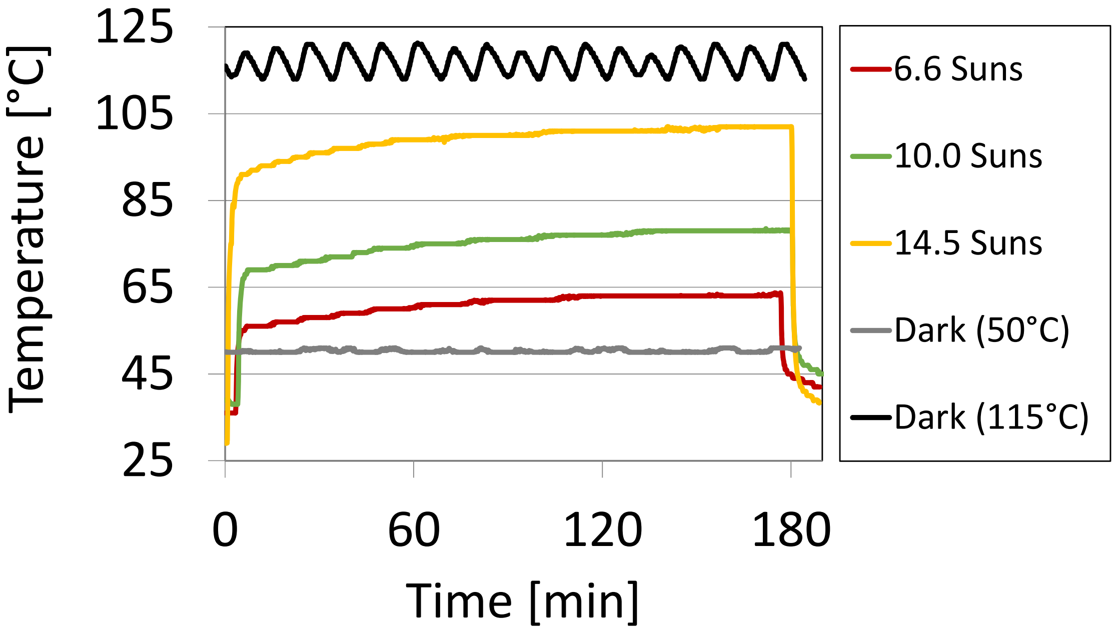

Figure S6 Temperature profiles of the Ru/SiNW sample for the batch reactions shown in Figure 3. The grey and black lines show the temperature recorded for tests carried out in the

dark at 50°C and 115°C, respectively. The red, green and yellow lines show the temperature scans carried out under solar simulated radiation at an intensity of 6.6, 10.0 and 14.5 suns.

[1] J. Garthwaite, “For Natural Gas-Fueled Cars, Long Road Looms Ahead” National Geographic, published September 4th, 2013.

[2] Eckle, S., Anfang, H., Behm, R. Reaction intermediates and side products in the methanation of CO and CO_2 over supported Ru catalysts in H_2-rich reformate gases. *J. Phys. Chem. C.* **115**, 1361-1367 (2011).

[3] L.B. Hoch, T. E. Wood, P. G. O’Brien, K. Liao, L. M. Reyes, C. A. Mims, G. A. Ozin, “The Rational Design of a Single-Component Photocatalyst for Gas-Phase CO_2 Reduction Using Both UV and Visible Light” (submitted for publication)

[4] ASTMG, 173–03, Standard Tables for Reference Solar Spectral Irradiances: Direct Normal and Hemispherical on 37 Degree Tilted Surface (ASTM International, 2005).
